# Matched analysis of induction chemotherapy plus chemoradiotherapy *versus* induction chemotherapy plus radiotherapy alone in locoregionally advanced nasopharyngeal carcinoma: a multicenter study

**DOI:** 10.18632/oncotarget.13285

**Published:** 2016-11-10

**Authors:** Bin Zhang, Ying Hu, Rui-Hua Xiong, Yu-Fei Pan, Qian-Lan Xu, Xiang-Yun Kong, Rui Cai, Qiu-Qiu Chen, Hua-Ying Tang, Wei Jiang

**Affiliations:** ^1^ Department of Radiation Oncology, Affiliated Hospital of Guilin Medical University, Guilin, PR China; ^2^ Department of Radiation Oncology, Wuzhou Red Cross Hospital, Wuzhou, PR China; ^3^ Department of Radiation Oncology, 181st Hospital of People's Liberation Army, Guilin, PR China; ^4^ Department of Radiation Oncology, Nan Xishan Hospital, Guilin, PR China

**Keywords:** nasopharyngeal carcinoma, induction chemotherapy, radiotherapy, concurrent chemoradiotherapy

## Abstract

**Background:**

The relative efficacy of induction chemotherapy (IC) followed by concurrent chemoradiotherapy (CCRT) versus IC followed by radiotherapy (RT) alone in locoregionally advanced NPC remains unclear.

**Methods:**

A total of 877 patients with locally advanced NPC who underwent IC/CCRT or IC/RT at four institutions in China between January 2004 and December 2010 were retrospectively assessed. IC was cisplatin-based combination chemotherapy; concurrent chemotherapy, single agent cisplatin. Two-dimensional conventional radiotherapy (2DCRT) was the radiotherapy technique. All patients were matched in an equal ratio using a pair-matched method. Overall survival (OS), disease-free survival (DFS), distant metastasis-free survival (DMFS), locoregional relapse-free survival (LRRFS) and toxicities were assessed.

**Results:**

Eligible patients were matched (*n* = 642; 321 patients per arm) based on eight clinicopathological characteristics. Five-year OS, DFS, DMFS, and LRRFS were 76%, 70%, 86%, and 88% for IC/CCRT and 75%, 70%, 90%, and 91% for IC/RT, respectively. There were no statistically significant survival differences between arms (*P*>0.05), even in subgroup analysis. In multivariate analysis, treatment (IC/CCRT *vs*. IC/RT) was not an independent prognostic factor for any survival end-point. Grade 3/4 acute gastrointestinal toxicities (vomiting, nausea) and hematological toxicities (leucopenia/neutropenia, thrombocytopenia and anemia) were significantly more common in the IC/CCRT arm than IC/RT arm during RT.

**Conclusion:**

Overall, IC/CCRT failed to demonstrate any survival advantage but higher acute toxicities over IC/RT in locoregionally advanced NPC.

## INTRODUCTION

Nasopharyngeal carcinoma (NPC) is an endemic malignant tumor that is highly prevalent in a number of regions, including southeast Asia and southern China [1]. As it is highly chemo- and radiosensitive, chemotherapy in combination with radiotherapy (RT) has become the standard recommended treatment in the NCCN guidelines for locoregionally advanced NPC [2].

Numerous studies have demonstrated that induction chemotherapy (IC) followed by concurrent chemoradiotherapy (CCRT) represents an effective approach to significantly improve survival in locoregionally advanced NPC [3, 4]. This strategy is widely applied in the areas of China where NPC is endemic. However, a high frequency of toxicities is observed during concurrent chemotherapy and 20%-40% of patients with NPC cannot complete their planned course of concurrent chemotherapy due to severe toxicities [5, 6]. Therefore, the optimal sequence of RT and chemotherapy in NPC remains to be clarified.

As far as we are aware, it is unclear whether the addition of concurrent chemotherapy to RT administered after IC improves the efficacy of treatment compared to IC/RT. Encouraging results for IC plus RT alone have been reported in clinical studies. Zeng et al. reported IC/RT provided good survival outcomes, with 5-year overall survival, cancer-specific survival, distant metastasis-free survival, and locoregional relapse-free survival rates of 62.1%, 65.2%, 88.2.4%, and 75.3% [7]. Wu et al. reported an equivalent 3-year overall survival rate of 86.8% for IC/RT, which was similar to the rate observed for CCRT in the same study [8]. In particular, as imaging techniques have developed, magnetic resonance imaging (MRI), 18F-fluorodeoxyglucose (FDG) positron emission tomography/computed tomography, (18F-FDG PET/CT) and high-resolution CT(HRCT) can provide more accurate target imaging, which has immensely improved the efficacy of radiotherapy alone in NPC [9,10]. It should be noted that similar survival outcomes were documented for the IC/RT and IC/CCRT arms in a small retrospective trial [11]. Therefore, the evidence in the literature suggests that IC followed by RT alone appears to be a feasible treatment strategy for locoregionally NPC.

At present, no high-quality randomised clinical trials have compared the efficacy of IC/RT and IC/CCRT in locoregionally advanced NPC, it is necessary to compare the efficacy and toxicities of IC followed by CCRT or RT alone in larger cohorts of patients. Therefore, we performed a large, retrospective, matched analysis to evaluate the treatment outcomes of IC/CCRT and IC/RT in locoregionally advanced NPC.

## RESULTS

### Baseline characteristics and treatment

The clinical data of the 877 patients with advanced NPC treated between January 2004 and December 2010 who met all of the criteria was registered. After matching based on eight clinicopathological characteristics, a total of 642 patients were included in this study: 321 received IC followed by RT alone and 321 received IC plus CCRT. The median patient age was relatively low (50 years-old, range 15 to 70 years-old), 498/642 (77.6%) patients were male and 144 /642 (22.4%) patients were female; 389/642 (60.6%) patients had stage III NPC, 153 /642 (23.8%), stage IVa, and 100/642 (15.6%), stage IVb. Baseline characteristics were well balanced between the two arms (Table 1).

**Table 1:** Clinicopathological characteristics of nasopharyngeal carcinoma patients with stage III-IVb

	Unmatched			Matched		
	IC/CCRT	IC/RT	*P*-value	IC/CCRT	IC/RT	*P*-value
	499 (56.9%)	378 (43.1%)		321(50.0%)	321(50.0%)	
Age (years)			0.059			0.269
≤ 45 years	262 (52.5%)	174 (46.0%)		151 (47.1%)	165 (51.4%)	
> 45 years	237 (47.5)%	204 (54.0%)		170 (52.9%)	156 (48.6%)	
Sex			0.696			0.256
Male	378 (75.8%)	282 (74.6%)		255 (79.4%)	243 (75.7%)	
Female	121 (24.2%)	96 (25.4%)		66 (20.6%)	78 (24.3%)	
T category			0.720			0.819
T1	15 (3.0%)	11 (2.9%)		13 (4.0%)	9 (2.8%)	
T2	75 (15.0%)	67 (17.7%)		56 (17.4%)	59 (18.4%)	
T3	283 (56.7%)	203 (53.7%)		167 (52.0%)	164 (51.1%)	
T4	126 (25.3%)	97 (25.7%)		85 (26.5%)	89 (27.7%)	
N category			0.006			0.438
N0	59 (11.8%)	35 (9.3%)		24 (7.5%)	25 (7.8%)	
N1	174 (34.9%)	143 (37.8%)		106 (33.0%)	114 (35.5%)	
N2	169 (33.9%)	155 (41.0%)		134 (41.7%)	140 (43.6%)	
N3	97 (19.4%)	45 (11.9%)		57 (17.8%)	42 (13.1%)	
Clinical stage			0.011			0.213
III	298 (59.7%)	245 (64.8%)		190 (59.2%)	199 (62.0%)	
IVa	104 (20.8%)	88 (23.4%)		74 (23.1%)	80 (24.9%)	
IVb	97 (19.4%)	45 (11.9%)		57 (17.8%)	42 (13.1%)	
Histological type			1.000			1.000
I	0 (0%)	0 (0%)		0 (0%)	0 (0%)	
II/III	499 (100%)	378 (100%)		321 (100%)	321 (100%)	
IC regimen			0.145			0.130
Cisplatin/fluorouracil	360 (72.1%)	279 (73.8%)		228 (71.0%)	238 (74.1%)	
Cisplatin/paclitaxel	94 (18.8%)	55 (14.5%)		67 (20.9%)	48 (15.0%)	
Other	45 (9.0%)	44 (11.6%)		26 (8.1%)	35 (10.9%)	
IC cycles			0.455			1.000
≤ 2 cycles	472 (94.6%)	353 (93.4%)		300 (93.5%)	300 (93.5%)	
> 2 cycles	27 (5.4%)	25 (6.6%)		21 (6.5%)	21 (6.5%)	

IC, induction chemotherapy; CCRT, concurrent chemoradiotherapy; RT, radiotherapy.

*P*-values were calculated using the Chi-square test, or Fisher's exact test if the expected number was < 5 in at least 25% of cells.

All 642 patients completed the prescribed course of two-dimensional conventional radiotherapy (2D-CRT). Of the 642 patients receiving IC, 128 patients completed one cycle of IC, 472 completed two cycles and 42 completed three or more cycles; 466/642 (72.6%) received the PF regimen and 115/642 (17.9%) received the PT regimen. In the IC/CCRT arm, 321 patients received concurrent chemotherapy; 58/321 (18%) received weekly administration of cisplatin and 263/321 (82%) received three courses of cisplatin, the median total dose of cisplatin was 240 mg/m^2^ (IQR 120-360). Overall, 102/321 (32%) patients could not complete three courses of concurrent chemotherapy during RT due to toxic effects.

### Response and survival outcomes

Three months after the completion of all treatment, treatment response was assessed for all 642 patients. The addition of concurrent chemotherapy to RT did not significantly increase the objective response rate (complete response and partial response; 97% *vs*. 97%, *P* =1.000).

**Figure 1:**
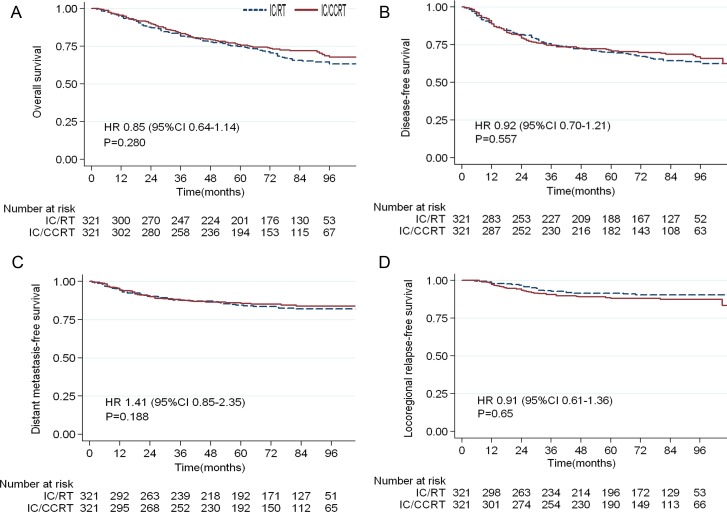
Kaplan-Meier survival curves for patients with locoregionally advanced nasopharyngeal carcinoma in the IC/CCRT and IC/RT arms **A**. overall survival; **B**. disease-free survival; **C**. distant metastasis-free survival; **D**. locoregional relapse-free survival.

The median follow-up duration for all patients was 73 months (range, 2-129 months). In the unmatched patients (*n* = 877), the 5-year OS, DFS, DMFS and LRRFS rates did not differ significantly between the IC/CCRT and IC/RT arms (OS, 77% *vs*. 75%, *P* = 0.148; DFS, 72% *vs*. 70%, *P* = 0.355; DMFS, 84% *vs*. 83%, *P* = 0.949; LRRFS, 89% *vs* 91%, *P* = 0.267). In the matched patients (*n* = 642), the 5-year overall survival rates for the IC/CCRT and IC/RT arms were 76% and 75%, respectively, with a hazard ratio of 0.72 (95% CI, 0.94 to 1.02). The corresponding 5-year DFS, DMFS, and LRRFS rates were 70%, 86%, and 88% for the IC/CCRT arm and 70%, 90%, and 91% for the IC/RT arm. Table 2 summarizes the survival outcomes of the two arms, and demonstrates the non-significant trends in favor of the addition of concurrent chemotherapy to IC/RT (Figure 1).

**Table 2:** Comparison of the survival rates for IC/CCRT *versus* IC/RT

Variable	IC/CCRT (*n* = 321)	IC/RT (*n* =321)	HR (95% CI)	*P*-value
Overall survival (%)			0.85 (0.64-1.14)	0.280
At 3-years	83	82		
At 5-years	76	75		
Disease-free survival (%)			0.92 (0.70-1.21)	0.557
At 3-years	73	74		
At 5-years	70	70		
Distant metastasis-free survival (%)			1.41 (0.85-2.35)	0.188
At 3-years	88	88		
At 5-years	86	90		
Locoregional relapse-free survival (%)			0.91 (0.61-1.36)	0.650
At 3-years	90	93		
At 5-years	88	91		

HR, hazard ratio; CI, confidence interval; IC, induction chemotherapy; CCRT, concurrent chemoradiotherapy; RT: radiotherapy.

### Subgroup analysis

Subgroup analyses were performed for T3-4 category, N2-3 category, IC regimen (PF or PT), and the number of IC cycles (≤ 2 or > 2 cycles). In the N2-3 category subgroup, concurrent chemotherapy seemed to provide an OS with a HR of 1.14 (95% CI: 0.81-1.60), however, this trend did not reach statistical significance (*P* = 0.465). In patients that received different IC regimens, concurrent chemotherapy tended to improve DFS, DMFS and LRRFS, though these effects were also not statistically significant (*P* > 0.05). Similarly, in the subgroup of IC cycles, no significant increase in any survival outcome was observed for patients who received concurrent chemotherapy (*P* > 0.05, Table 3).

**Table 3:** Subgroup analysis of IC/CCRT *versus* IC/RT by tumor stage and induction chemotherapy regimen

Subgroup	Overall survival		Disease-free survival		Distant metastasis-free survival		Locoregional relapse-free survival	
	HR (95% CI)	*P*	HR (95% CI)	*P*	HR (95% CI)	*P*	HR (95% CI)	*P*
T, N stage								
T3-4	0.91 (0.65-1.26)	0.556	0.92 (0.67-1.26)	0.583	1.00 (0.64-1.58)	0.996	1.03 (0.54-1.99)	0.921
N2-3	1.14 (0.81-1.60)	0.462	1.23 (0.88-1.71)	0.233	1.33 (0.82-2.18)	0.254	1.68 (0.91-3.12)	0.100
IC regimen								
PF	0.59 (0.30-1.15)	0.121	0.56 (0.29-1.07)	0.079	0,52 (0.20-1.31)	0.165	0.51 (0.15-1.66)	0.259
PT	0.93 (0.67-1.30)	0.675	1.02 (0.74-1.40)	0.911	1.01 (0.63-1.61)	0.973	1.68 (0.90-3.15)	0.105
IC cycles								
≤ 2 cycles	0.85 (0.63-1.15)	0.284	0.91 (0.69-1.21)	0.528	0.91 (0.60-1.37)	0.649	1.45 0.85-2.46()	0.172
> 2 cycles	0.93 (0.33-2.65)	0.893	0.83 (0.33-2.65)	0.893	1,44 (0.24-8.60)	0.692	0.46 (0.04-5.09)	*0.528*

HR, hazard ratio; CI, confidence interval; PF: cisplatin/fluorouracil; PT: cisplatin/paclitaxel; IC, induction chemotherapy.

### Prognostic factors

Multivariate analysis was performed to further assess prognostic factors. Various potential prognostic factors for OS, DFS, DMFS and LRRFS, including sex, age, T category, N category, and treatment arm were evaluated. In multivariate analysis, T and N category were identified as significant prognostic factors for OS, DFS, DMFS, and age was an independent prognostic factor for OS (Table 4). Sex, age and treatment arm were not significant prognostic factors for any survival outcome.

**Table 4:** Multivariate analysis of the associations between significant prognostic factors and survival outcomes in the propensity-matched cohort

Outcome	Hazard ratio	95% CI	*P*-value
Overall survival			
Sex female vs. male	0.73	0.50-1.06	0.098
Age > 45 vs. ≤ 45 years	1.47	1.10-1.97	0.009
T category T1-2 vs.T3-4	1.50	1.23-1.82	0.000
N category N0-1 vs. N2-3	1.72	1.43-2.08	0.000
Treatment arm IC/CCRT vs. IC/RT	0.87	0.65-1.16	0.331
Distant-free survival			
Sex female vs. male	0.70	0.48-1.00	0.051
Age > 45 vs. ≤ 45 years	1.30	0.99-1.71	0.064
T category T1-2 vs.T3-4	1.38	1.14-1.67	0.001
N category N0-1 vs. N2-3	1.62	1.36-1.93	0.000
Treatment arm IC/CCRT vs. IC/RT	0.93	0.71-1.22	0.597
Distant metastasis-free survival			
Sex female vs. male	0.60	0.34-1.06	0.080
Age > 45 vs. ≤ 45 years	1.38	0.92-2.08	0.119
T category T1-2 vs.T3-4	1.51	1.14-2.00	0.004
N category N0-1 vs. N2-3	1.82	1.40-2.38	0.000
Treatment arm IC/CCRT vs. IC/RT	0.92	0.62-1.38	0.688
Locoregional relapse-free survival			
Sex female vs. male	0.74	0.39-1.43	0.373
Age > 45 vs. ≤ 45 years	0.93	0.56-1.54	0.785
T category T1-2 vs.T3-4	0.86	0.61-1.22	0.398
N category N0-1 vs. N2-3	1.25	0.89-1.75	0.193
Treatment arm IC/CCRT vs. IC/RT	1.36	0.82-2,27	0.238

IC, induction chemotherapy; CCRT, concurrent chemoradiotherapy; RT: radiotherapy; CI, confidence interval.

### Toxicities

No treatment-related deaths were observed in either arm. Overall, in terms of acute toxicities during IC, we noted no significant differences between the two arms (*P* > 0.05). Regarding acute toxicities during RT, grade 3-4 adverse events appeared to be more frequent in the IC/CCRT arm than the IC/RT arm. For example, the most common gastrointestinal events were nausea/vomiting (24% *vs*. 3%, *P* = 0.000) and mucositis (49% *vs*. 30%, *P* = 0.000), and the most frequent hematological toxicities were leucopenia/neutropenia (12% *vs*. 1.9%, *P* = 0.000), thrombocytopenia (9% *vs*. 1.2%, *P* = 0.000) and anemia (3.7% *vs*. 0.6%, *P* = 0.015). In analysis of grade 3-4 late toxicities, the frequencies of cranial neuropathy, temporal lobe necrosis, ear problems (deafness/otitis) and neck tissue damage were similar between the IC/CCRT and IC/RT arms (*P* > 0.05; Table 5).

**Table 5:** Profile of treatment-related toxicities

	IC/CCRT (*n* = 321)	IC/RT (*n* = 321)	*P*
Grade 3-4 adverse events during IC, *n* (%)			
Hematological			
Leukopenia/neutropenia	100 (31%)	106 (33%)	0.672
Thrombocytopenia	16 (5%)	13(4%)	0.704
Anemia	13(4%)	10 (3%)	0.671
Non-hematological			
Stomatitis (mucositis)	5(1.6%)	3(1%)	0.722
Nausea/ vomiting	55(17%)	48(15%)	0.519
Diarrhea	8 (2.5%)	11 (3.5%)	0.641
Liver dysfunction	3(1%)	2(0.6%)	1.000
Kidney dysfunction	0	0	1.000
Grade 3-4 adverse events during RT, *n* (%)			
Hematological			
Leukopenia/neutropenia	39(12%)	6(1.9%)	0.000
Thrombocytopenia	28(9%)	4(1.2%)	0.000
Anemia	12(3.7%)	2(0.6%)	0.015
Non-hematological			
Skin reaction (radiation-related)	22(7%)	16(5%)	0.403
Mucositis (radiation-related)	157(49%)	96(30%)	0.000
Nausea /vomiting	77(24%)	9(3%)	0.000
Dry mouth	102(32%)	87(27%)	0.225
Grade 3-4 late toxicities, *n* (%)			
Cranial neuropathy	14 (4.5%)	16 (5%)	0.708
Temporal lobe necrosis	22 (7%)	29 (9%)	0.307
Ear (deafness/otitis)	132 (41%)	119(37%)	0.293
Neck tissue damage	80 (25%)	67 (21%)	0.222

IC, induction chemotherapy; CCRT, concurrent chemoradiotherapy; RT: radiotherapy.

*P*-values were calculated using the Chi-square test (or Fisher's exact test if the expected number was < 5 in at least 25% of cells).

## DISCUSSION

This retrospective matched analysis assessed a relatively large cohort of patients with locally advanced NPC (*n* = 642) in order to further compare the survival outcomes and toxicities of IC followed by RT alone or CCRT. Although IC followed by CCRT has emerged as the current standard treatment for locoregionally advanced NPC according to the NCCN guidelines [2], the present study indicates the addition of concurrent chemotherapy does not translate to any significant survival advantage over IC/RT. Moreover, IC/CCRT appears to increase the risk of toxicities.

Both induction and concurrent chemotherapy have been proposed as effective treatment strategies for NPC [12–14]. However, the most beneficial sequence of chemotherapy for patients has not yet been established. Lin et al. reported the advantages of IC plus RT in terms of 3-year local control and overall survival (95%, 89%) in NPC [15]. Additionally, a meta-analysis demonstrated IC followed by RT reduced the locoregional recurrence rate (LRR) and distant metastasis rate (DMR) and improved overall survival (OS) in NPC [16]. In a long-term outcome report [17], IC followed by RT resulted in excellent 10-year overall survival and failure-free survival rates of 49.5% and 48%. In the current study, IC plus RT also provided superb outcomes in terms of 5-year overall survival (75%) and disease-free survival (70%) in patients with locoregionally advanced NPC.

A prior study conducted by Huang et al. [6] administered chemotherapy concurrently with radiotherapy after IC in the hope of achieving survival benefits. Unfortunately, the addition of concurrent chemotherapy did not result in superior survival outcomes compared to IC/RT. Indeed, several trials have reported the same outcomes for IC/RT and IC/CCRT. The small retrospective study conducted by Li et al. demonstrated that IC/CCRT did not provide any survival benefit over IC/RT (*P* > 0.05) [11]. In the trial conducted by Su et al., additional concurrent chemotherapy did not provide a significant OS benefit over IC/RT (82.3% *vs*. 73.4%, *P* > 0.05) in locally advanced NPC [18]. In this study, we also failed to observe a better outcome for IC/CCRT over IC/RT in terms of either 5-year OS (76% *vs*. 75%, *P* = 0.280) or DFS (70% *vs*. 70%, *P* = 0.557). Evaluation of prognostic factors using multivariable analysis demonstrated concurrent chemotherapy was also not an independent factor affecting survival. Furthermore, subgroup analysis did not indicate significant prognostic value for concurrent chemotherapy. Therefore, the available evidence indicates additional concurrent chemotherapy negatively affects treatment, rather than providing a survival benefit.

Compared to concurrent chemotherapy, IC has several potential advantages. Firstly, early use of chemotherapy drugs at full dose before RT enables better drug delivery through the vasculature and can effectively eradicate micrometastases. Secondly, IC can be better tolerated by patients at the initial stage of treatment, which leads to better compliance. Finally, IC is administered to shrink the primary tumor and increase tumor radio-sensitivity [19, 20]. Simultaneously, a favorable increase in locoregional control is achieved with concurrent chemotherapy; however, its ability to control distant metastasis is relatively limited, as proven in numerous studies [21, 22]. Additionally, with the development of imaging techniques, MRI, 18F-FDG PET/CT and HRCT are recommended as the preferred modalities for staging patients with NPC [9, 10]. Likewise, the gross tumor volume (GTV) is defined more accurately during radiotherapy planning for 2D-CRT, which provides significant value in terms of locoregional control in NPC. Notably, further improvement in locoregional control by the addition of concurrent chemotherapy is meaningless. Therefore, based on our results, we do not recommend the addition of concurrent chemotherapy to RT after IC in locally advanced NPC.

Existing data indicates drug-related toxicities induced by CCRT lead to the interruption of RT or concurrent chemotherapy in many patients with NPC [23,24]. The current study reveals additional concurrent chemotherapy increases the frequency of grade 3-4 toxicities, especially vomiting, nausea, leukopenia, thrombocytopenia and anemia, in agreement with the results of a phase III randomized trial reported by Huang et al. [6]. Due to these excess toxicities, the tolerance of patients to concurrent chemotherapy during RT was usually very poor and compliance was 68% in this study, in accordance with other studies [25].

There are several limitations to this study. First, this was a retrospective analysis; therefore, selection bias could not be avoided, although pair-matched analysis can reduce selection bias. Secondly, standardized protocols and chemotherapy strategies were not employed for the entire cohort. Thirdly, 2D-CRT was used as the radiation technology; this technique is proven to result in a lower locoregional control rate than IMRT [26]. Therefore, the results of this study require validation in phase III prospective trials of patients with locally advanced NPC treated using IMRT.

In conclusion, this analysis failed to demonstrate the superiority of IC/CCRT over IC/RT in locoregionally advanced NPC, with concurrent chemotherapy contributing to higher frequencies of toxicities. In light of the wide-spread adoption of advanced RT technologies, we plan to conduct a prospective trial address the true value of IC/CCRT versus IC/RT in patients with locally advanced NPC treated using IMRT.

## MATERIALS and METHODS

### Participants and study design

A total of 1697 NPC patients who received IC between January 2004 and December 2010 were retrospectively accrued from four radiation oncology institutions: the Affiliated Hospital of Guilin Medical University, Wuzhou Red Cross Hospital, 181st Hospital of People's Liberation Army, and Nanxishan Hospital. The inclusion criteria were newly pathologically-diagnosed, non-distant metastatic stage III-IVb NPC according to the 7th edition of American Joint Committee on Cancer (AJCC) or Union for International Cancer Control (UICC) staging system [27]; age 15-70 years-old; and normal complete blood count, normal hepatic and renal function and a Karnofsky Score ≥ 70. The exclusion criteria were: age > 70 years-old (*n* = 9), stage I or stage II NPC (*n* = 151), patients who received adjuvant chemotherapy (*n* = 584) and those with insufficient data (*n* = 76). Ultimately, 877 patients with locoregionally advanced NPC who had been treated with IC/RT or IC/CCRT were included. The two arms were matched for eight clinicopathological characteristics [28]: age (≤ 45 years *vs*. > 45 years), sex (male *vs*. female), T category (T1 *vs*. T2 *vs*. T3 *vs*. T4), N category (N0 *vs*. N1 *vs*. N2 *vs*. N3), clinical stage (III *vs*. IVa *vs*. IVb), histological type (type I *vs*. type II/III), IC regimen (cisplatin/fluorouracil *vs*. cisplatin/paclitaxel *vs*. other) and number of IC cycles (≤ 2 *vs*. > 2 cycles). After matching, 642 patients were classified into one of the two arms: IC followed by RT or IC followed by CCRT, with 321 patients in each arm. Overall survival (OS), disease-free survival (DFS), distant metastasis-free survival (DMFS), locoregional relapse-free survival (LRRFS) and treatment related toxicities were compared between the two arms.

The study was conducted in accordance with the Declaration of Helsinki, Good Clinical Practice Guidelines and national and international guidelines, and was approved by the Research Ethics Committees of all four institutions. All patients provided written informed consent.

### Treatment

All patients underwent two-dimensional conventional radiotherapy (2D-CRT) delivered by a linear accelerator device including 6 million Volt photons. The primary nasopharyngeal tumor was irradiated with a dose of 68-74 Gy. The metastatic lymph nodes in the cervical region were prescribed a dose of 66-70 Gy and the cervical region without lymph nodes metastases a dose of 50-54 Gy. All patients were treated with 2.0 Gy per fraction daily, 5 days per week. During the period of IC, 466 patients received cisplatin (25 mg/m^2^, on day 1-3) and 5-fluorouracil (600-800 mg/m^2^, on days 1-5), 115 patients received cisplatin (25 mg/m^2^, on day 1-3) and paclitaxel (135 mg/m^2^, on day 1), and 61 patients received other regimens (such as cisplatin plus docetaxel or cisplatin plus gemcitabine). All regimens were repeated every 3 weeks for one or more cycles. After IC, all patients were treated with 2D-CRT. The study-defined CCRT regimen was 30 mg/m^2^ cisplatin on day 1 per week for 6 cycles or 75 mg/m^2^ cisplatin on day 1 every 3 weeks for 3 cycles.

### Assessments and follow up

Pretreatment assessments included a physical examination, chest CT, electrocardiogram, liver ultrasound, dental examination, and other laboratory investigations such as a complete blood cell count and full biochemical profiles. Nasopharyngeal fiber optic endoscopy and biopsy, and MRI or CT of the nasopharynx and neck were performed at baseline. Patients with bone pain underwent chest and abdominal CT scans and bone ECT scans for metastatic workup.

Clinical evaluation of efficacy of treatment was assessed according to the Response Evaluation Criteria in Solid Tumors (RECIST) version 1.0 [29]. Acute treatment-related toxicities (occurring from the start of IC to three months after RT) were evaluated based on the Common Terminology Criteria for Adverse Events (CTCAE) version 3.0 [30]. Late RT-related toxicities (occurring or persisting beyond three months from initiation of RT) were graded according to the Radiation Morbidity Scoring Criteria of the Radiation Therapy Oncology Group. Follow-up was measured from the first day of treatment to the last day of examination or death. During the follow-up period, endoscopic biopsy and MRI or CT of the nasopharynx and neck were performed to diagnose all locoregional recurrences and irresolute cases were diagnosed by fine-needle aspiration. Distant metastasis was confirmed by chest and liver CT scans or bone emission computed tomography scans. All patients were assessed every 3 months for the first 3 years, every 6 months in the fourth and fifth years and annually thereafter.

### Endpoints and statistical analysis

The primary endpoint was overall survival (OS, from initiation of treatment to death of any cause or last follow-up). Secondary clinical endpoints included disease-free survival (DFS, from initiation of treatment to first disease progression [local recurrence and/or distant metastasis] or death from any cause), distant metastasis-free survival (DMFS, from initiation of treatment to first distant metastasis), locoregional relapse-free survival (LRRFS, from initiation of treatment to locoregional progression), and treatment-related toxicities.

Clinical characteristics and treatment-related toxicity rates were evaluated using Fisher's exact test. The Kaplan-Meier method was used to estimate survival rates and the log-rank test was used to compare the survival distributions. Cox proportional hazards model was employed to identify prognostic factors associated with survival outcomes and estimate hazard ratios (HR) using the backward stepwise method. The potentially significant prognostic factors included the following: sex, age, T category, N category and treatment strategies. All statistical analyses were carried out using Stata version 13.0 (StataCorp LP, College Station, Texas, USA). Two-tailed *P*-values < 0.05 were considered statistically significant.
